# The Cannabis Proteome Draft Map Project

**DOI:** 10.3390/ijms21030965

**Published:** 2020-01-31

**Authors:** Conor Jenkins, Benjamin Orsburn

**Affiliations:** 1 Department of Biology, Hood College, Frederick, MD 21701, USA; conor.jenkins@outlook.com; 2 Think20 Labs, Columbia, MD 21046, USA; 3 Department of Core Facilities, University of Virginia School of Medicine, Charlottesville, VA 22908, USA

**Keywords:** Proteomics, proteogenomics, Cannabis, PTMs

## Abstract

Recently we have seen a relaxation of the historic restrictions on the use and subsequent research on the *Cannabis* plants, generally classified as *Cannabis sativa* and *Cannabis indica*. What research has been performed to date has centered on chemical analysis of plant flower products, namely cannabinoids and various terpenes that directly contribute to phenotypic characteristics of the female flowers. In addition, we have seen many groups recently completing genetic profiles of various plants of commercial value. To date, no comprehensive attempt has been made to profile the proteomes of these plants. We report herein our progress on constructing a comprehensive draft map of the *Cannabis* proteome. To date we have identified over 17,000 potential protein sequences. Unfortunately, no annotated genome of *Cannabis* plants currently exists. We present a method by which “next generation” DNA sequencing output and shotgun proteomics data can be combined to produce annotated FASTA files, bypassing the need for annotated genetic information altogether in traditional proteomics workflows. The resulting material represents the first comprehensive annotated protein FASTA for any *Cannabis* plant. Using this annotated database as reference we can refine our protein identifications, resulting in the confident identification of 13,000 proteins with putative function. Furthermore, we demonstrate that post-translational modifications play an important role in the proteomes of *Cannabis* flower, particularly lysine acetylation and protein glycosylation. To facilitate the evolution of analytical investigations into these plant materials, we have created a portal to host resources developed from our proteomic and metabolomic analysis of *Cannabis* plant material as well as our results integrating these resources.

## 1. Introduction

Proteomics is a science dedicated to the creation of comprehensive quantitative snapshots of all the proteins produced by an individual organism, tissue or cell [1]. The term was coined in the 1990s during the efforts to sequence the first complete human genomes [2]. While the technology was in place to complete the human genome draft in 2003, the first two drafts of the human proteome were not completed by teams led by Johns Hopkins and Center for Integrated Protein Science Munich researchers until 2014. These two separate and ambitious projects were the composite of thousands of hours of instrument run time utilizing the most sophisticated hardware available at that time [3,4]. Recent advances in mass spectrometry technology now permit the completion of proteome profiles in more practical time. Single celled organisms have been “fully” sequenced in less than an hour, and by use of multi-dimensional chromatography, relatively high coverage human proteomes have been completed in only a few days [5,6,7]. While much can be learned by sequencing DNA and RNA in a cell, quantifying and sequencing the proteome has distinct advantages as proteins perform physical and enzymatic activities in the cell and linking them more directly to the phenotype [8]. RNA sequencing may correctly predict the presence and relative abundance of proteins, but the variance in translation and degradation rates, as well as the proteins’ ability to be inactivated by chemical modifications, may make predictions of function from RNA abundance data wholly inaccurate. Furthermore, many proteins are altered by chemical post-translational modifications such as phosphorylation and acetylation which may completely change the protein function by serving as on/off switches for motion or metabolism [9,10]. Protein modifications are directly involved in nearly every known disease and these modifications are impossible to identify with any current DNA/RNA sequencing technology [11].

In North America we have recently witnessed an alleviation of restrictions on the use and subsequent research of plants belonging to the *Cannabis* genus. To date, relatively little work has been performed on these plants in any regard and no comprehensive study of the proteome has ever been attempted. In a study published in 2004, Reharjo et al., described a differential proteomics approach for the studying of *Cannabis sativa* plant tissues. Differential analysis was performed by two-dimensional gel electrophoresis (2D-gel), followed by mass spectrometry. The counting of gel spots indicated at least 800 proteins were present in these tissue, but due to technological restraints of that time, less than 100 were identified [12].

We report herein the methodology and preliminary results of our attempts to create the first draft map of the *Cannabis* proteome. Proteins were extracted from plant tissue from stems and leaves of plants as well as from medical flower products from *C.sativa* and *C.indica* strains with well characterized cannabinoid profiles. Extracted proteins were digested, separated by ultrahigh pressure liquid chromatography and analyzed by high resolution tandem mass spectrometry. Data assembly on the high-resolution spectra has been completed, resulting in the annotation of over 17,000 potential protein coding regions. All data for this project is available to view or download at www.CannabisDraftMap.Org.

## 2. Results and Conclusion

### 2.1. Peptide and Protein Identifications

The lack of annotated genetic sequences and derived theoretical protein sequences is a considerable challenge for traditional proteomics workflows, which rely heavily on these resources for spectral matching. Using the custom proteogenomic workflow described here, we were able to identify a small percentage of the first 2.5 million MS/MS spectra obtained and match those to a compiled and in-house generated theoretical protein sequence database of greater than 41 million entries. This pipeline resulted in 135,845 peptide spectral matches, or approximately 5.4% identification rate. A recent quantitative study of Arabidopsis plant material using similar instrumentation as employed here demonstrated an identification rate of approximately 22% against the manually curated UniProt database for this plant. This result is unsurprising given the body of work that has been assembled for this model organism and suggests that further work will be necessary to refine our genomic and proteomic tools for the less studied *Cannabis* plant [13].

**Figure 1:**
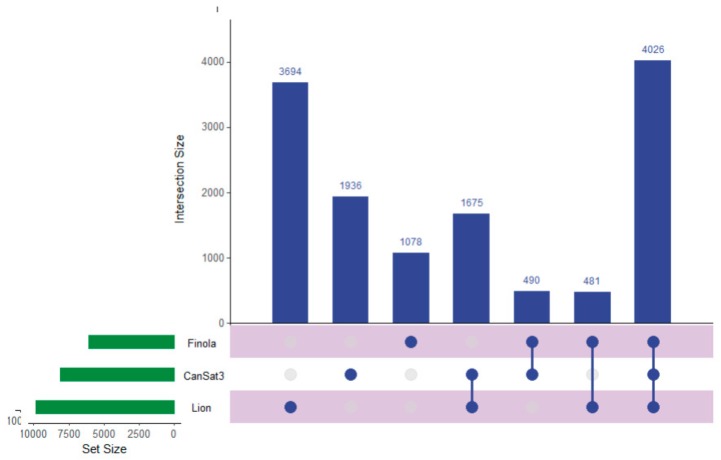
An UpSetR graph showing the unique protein identifications associated with each genomic dataset used for the generation of the proteogenomic FASTA as well as the number of proteins shared between the datasets.

**Figure 2:**
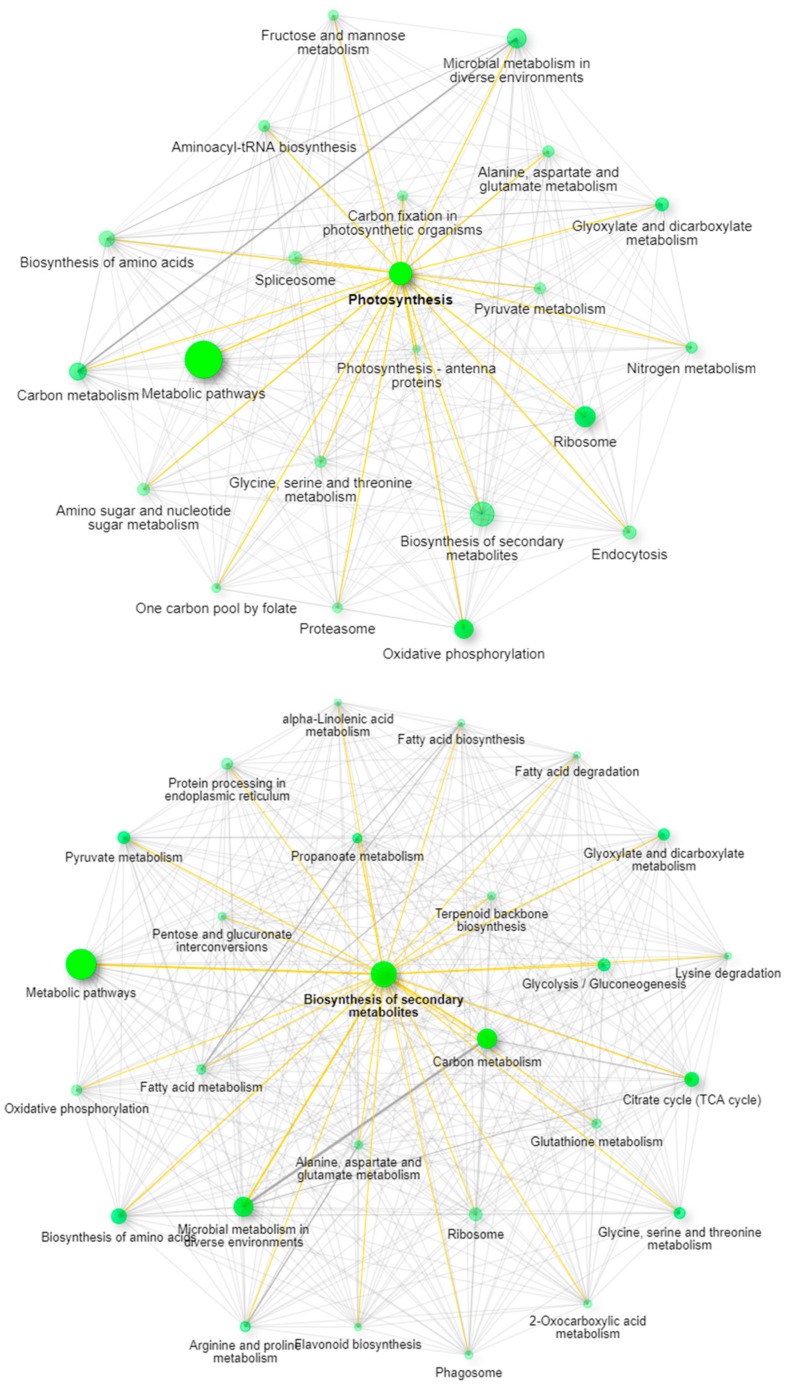
ShinyGO network analysis demonstrating the pathway differences between leaves (top panel) and mature female flowers (bottom panel).

Recent work has described the improvement and correction of genome annotations using high resolution mass spectrometry [14]. While this is beyond the scope of this study, we can develop metrics related to the quality of match of genomic data using high coverage proteomics. An UpSetR graph [15] is provided as Figure 1 that shows the unique protein identifications and matches to the various genomic databases both unique and conserved. To further illustrate the importance of an annotated cannabis protein database, containing the protein groups identified by the initial analysis, only 1838 representing less than 10% had sequence homology suitable to be matched against the UniProt/SwissProt database, despite the fact a database containing all manually curated protein sequences from green plants in UniProt was utilized in this work. A summary of these results is available as Supplemental Table S2. However, utilization of the UniProt/SwissProt Viridiplantae database does allow some insight into these plant materials. By utilizing the identified proteins from this database and relative quantification for gene ontology analysis with the recently described ShinyGO software [16] broad patterns emerge. Figure 2 is an example of these results when comparing the proteins most differentially expressed between male leaf plant material and a female flower.

When excluding the generic network for metabolic pathways, the most prevalent pathway node in leaves is photosynthesis, while in the flowering material the biosynthesis of secondary metabolites is the most obvious network node. The well-established pathways leading to the synthesis of cannabinoids in the plant feature prominently in this latter pathway with strong branching to both terpenoid backbone biosynthesis and fatty acid biosynthesis demonstrating multiple links within the network.

Despite these challenges we have built considerably on the existing knowledge of the *Cannabis* proteome and now have evidence for the expression of over 17,000 protein coding regions of the approximately 25,000 currently predicted [17]. Further work will be necessary to increase the number of annotated proteins and to validate the presence of the ones we have described herein.

A primary goal of this work was the development of tools to enable further proteomic analysis of *Cannabis* plant materials. Tools such as a comprehensive protein FASTA for the plant were required in order to move forward with this study and to investigate the materials we had acquired. In a study described elsewhere, traditional genomics pipelines were utilized to create a second protein FASTA database [18] and both these resources are available for other researchers to perform proteomic investigations in *Cannabis* plants, by downloading on the portal described in this work. We have also compiled all of our results and created spectral libraries in the commonly used Skyline library format. These tools will enable both targeted and data-independent acquisition experiments that were not possible before the creation of these tools.

Following the creation of the FASTA database, we reprocessed all mass spectrometry files using the FASTA as reference and obtained relative quantification of these proteins between the two datasets. The Scaffold output sheet for the green plant material analysis is available as Supplemental Table S6, while the same output for the flower specific analysis is provided as Supplemental Table S7.

### 2.2. Post-Translational Modification Identification and Analysis

The importance of protein post-translational modifications (PTMs) in Cannabis are, to our knowledge, relatively unknown, outside of results suggesting glycosylation on the THCA synthase protein [19]. Current strategies for identifying PTMs from shotgun proteomics data require the addition of dynamic modifications. Each single dynamic modification results in a doubling of the number of theoretical peptides and due to the presence of multiple modifications sites per proteins, indiscriminate searching of PTMs results in exponential increase in both the search space and required computational power to complete data processing [20]. To address these issues, we generated a new FASTA database that contained only the 17,269 proteins identified in our SEQUEST and Percolator searches of all high-resolution files. Using this newly reduced database of proteins that appear to be present and the complete theoretical sequences from these entries extracted from our original FASTAs, we can search these identified proteins for PTMs. For this analysis we chose to employ the recently described MetaMorpheus (MM) software. MM performs a tiered search strategy that is reliant on the recalibration of MS spectra and the Global Post-translational Modification Discovery (GPTMD) algorithm [21]. This next generation search engine can identify and quantify hundreds of unknown post-translational modifications with annotated databases on standard desktop computers [22,23]. Supplemental Table S3 contains a summary of these results. MM identified 26,477 unique peptides and 6111 PTMs in these files alone. The most common identified modification was methionine oxidation, which is often a product of the sample preparation process. Lysine acetylation appeared in a high number of PSMs and phosphorylation of serine and threonine were also observed.

To further confirm, localize and visualize these potential PTMs, the files were reanalyzed with MSAmanda 2.0 and the ptmRS software. The MSAmanda search engine was specifically designed for high resolution accurate MS/MS spectra and has been demonstrated to be a particularly powerful in the confident identification of PTMs. The ptmRS algorithm provides probability scoring of PTM site localization within the peptide chain and is most useful when multiple amino acid residues may host this chemical modification. MSAmanda 2.0 is a recent iteration of the software which allows far more practical sequencing speeds on standard desktop computers. Data visualization was performed using the recently described MS2Go software (www.pd-nodes.org). The complete MS2Go output is available for download at www.CannabisDraftMap.org under the Full Data Sets page.

**Figure 3:**
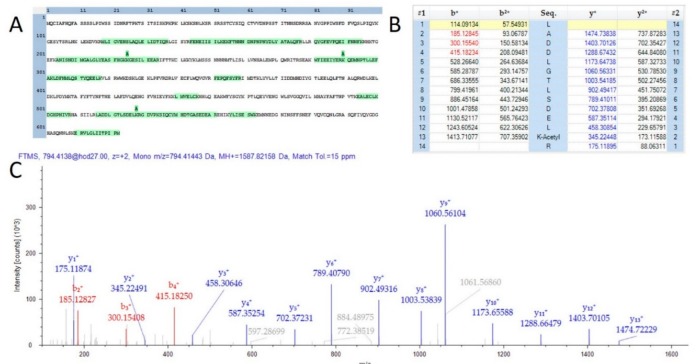
Evidence of acetylation on TPS1. (**A**) A sequence map demonstrating 3 observed lysine acetylation modifications. (**B**) A fragment map showing 100% sequence coverage for one acetylation site. (**C**) MS/MS spectra matching the fragment map.

A total of 584 proteins were identified that possessed at least one lysine acetylation within their protein sequence. The MS2Go output displaying these sequences is presented as Supplemental Table S5. Lysine acetylation has recently been described as a key modulator of the model organism *Arabidopsis thaliana* [24]. One interesting observation was that over 90% of the observed acetylation sites were unique to mature flowers and appeared entirely absent in the proteins of leaves and stems from the male plant materials analyzed in this study. Further analysis with a greater number of samples will be necessary to determine if this observation is an artifact of the extraction process for these very different plant materials. Lysine acetylation sites were observed in proteins involved in the production of compounds of commercial interest. Supplemental Figure S3 is a visualization of the sequence coverage and acetylation sites of the THCA synthase protein (THCAS), which is the key enzyme in the production of the molecule. Furthermore, three acetylated peptides were found in the TPS1 gene product which is involved the final stages of the synthesis of limonene. Figure 3 is a depiction of one peptide from this protein of interest. While of questionable medicinal value [25], the characteristic limonene fragrance is of specific interest to some consumers and the state of California requires the quantification of this terpene in all Cannabis products.

In addition to peptide sequence coverage, HCD fragmentation of lysine acetylated peptides typically results in the production of a diagnostic fragment ion of 126.0913 m/z [26]. All peptides demonstrated as acetylated in THCAS and TPS1 were further supported by manual validation of the presence of this diagnostic ion, although not within the scale of the image for the spectra chosen for TPS1. Further investigation with chemical enrichment of acetylated peptides will be required to determine the relative importance of this PTM in the flower and the production of secondary metabolites of commercial and medical value.

A previous study suggested the possible existence of glycosylation on THCAS at up to four sites. This conclusion was based on the presence of an unexpected gel shift during electrophoretic migration of purified protein and the presence of structural domains compatible with glycosylation [27]. Given the relatively high abundance of this protein, we found it surprising that few glycopeptides were predicted by the MetaMorpheus analysis. To further investigate we developed a new algorithm to search for evidence of glycosylation by searching spectra specifically for glycopeptide specific fragments.

We have recently described the Reporter Ion Data Analysis Reduction (R.I.D.A.R.) software [20], and it’s capability of removing spectra that are not quantitatively interesting to the end user from large shotgun proteomics datasets prior to database searching steps. By reducing the data in this manner, we can lower the processing time of large cohort studies to be manageable for processing on standard consumer level computer equipment. Diagnostic Ion Data Analysis Reduction (D.I.D.A.R.) is an extension of this logic and will be described in depth elsewhere. The python script allows an end user to create files that only contain spectra that exhibit a fragment ion or ions of specific interest [26]. It is well established in the literature that the HCD fragmentation of peptides with glycan moieties produce low mass oxonium ions [28,29].

We used the D.I.D.A.R. script to create a file containing only spectra possessing common diagnostic ions, including the HexNaC oxonium ions, 168 and 204 [30]. The diagnostic ions as well as the number of MS/MS spectra containing these markers is provided as Supplemental Table S12. Over 3 × 10^5^ MS/MS spectra returned as positive for glycan specific fragment ions, suggesting that nearly 10% of all ions fragmented in this study were glycosylated. To verify the validity of this output, we used the Xcalibur software to display an extracted ion chromatogram plotting only the signal of ions present in MS/MS spectra with an m/z within 5ppm of the HexNaC diagnostic fragment ion. This visualization is shown in Supplemental Figure S4. Further analysis will be necessary to characterize the glycoproteome of *Cannabis* materials and the relative importance of these seemingly common modifications in the regulation of the plant biology.

### 2.3. Utilizing this Resource toward the Meta-Analysis of Previous Studies

A recent study from Vincent et al., sought to develop an optimized protocol to study Cannabis proteomics [31]. This study utilized a linear ion trap Orbitrap (LTQ Orbitrap Velos) system and nanoflow HPLC system, an ultimately similar system to those employed in this work. While the focus of the work was digestion efficiency, the number of proteins identified were low by comparison, with less than 200 total protein identifications. The authors point out that this is due to the small number of annotated proteins present in the UniProt database. 

We reanalyzed the instrument vendor files from this work using the eggNOG FASTA databases developed in this study. The complete output file is available in Supplemental Table S9. Using our UniProt derived Viridiplantae database, we find that this work matches 330 confident protein groups (in sum). By adding the eggNOG FASTA database to this identical analysis we obtain a total of 2026 high confidence unique protein groups from the combination of all deposited instrument vendor files. 

### 2.4. Correlation Analysis between Small Molecules and Proteins

Correlation analysis in proteomics has been shown to be a powerful tool in the identification of cooperating proteins in biological processes [32]. We have recently described the identification and quantification of over 1000 small molecule features in medicinal products [33]. While the pathways leading to the production of the major cannabinoids has been the focus of intense study, little is known regarding the production of other central and secondary metabolites. The lists of small molecules and proteins with quantification values were combined resulting in correlation scores and significance values linking all metabolites to confidently identified and quantified proteins. The results of this analysis are presented in Supplemental Table S10.

**Figure 4:**
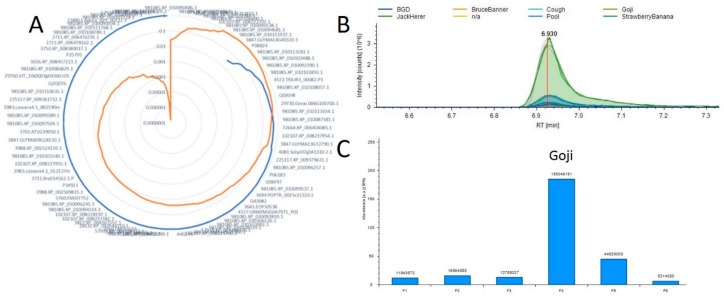
Example correlation analysis plot (**A**) Radar diagram for 11-OH-THC where the blue line represents all positive Pearson Correlation and orange is the p-value for each measurement. (**B**) A plot overlaying the 11-OH-THC metabolite peaks and technical replicates. (**C**) A plot of the protein from A demonstrating the highest correlation with this metabolite.

Using this tool as a starting point we hope to map all metabolic pathways in *Cannabis* plants as well as to identify both new molecules of interest and the proteins responsible for their production. Current work is focused on the acquisition of additional and more varied samples to further develop this resource. Figure 4 is an example of these results and the visualization provided by this tool. In this example we evaluate the 11-OH-THC molecule which appears to have a strong differential expression in one of the strains evaluated in both studies. We found approximately 85 proteins that possessed a positive correlation across the 6 plant materials with the relative abundance of this molecule, and the full list is presented in Supplemental Table S11. The protein exhibiting both the highest Pearson correlation (0.9997) and corresponding p-value (1.54 × 10^−7^) was annotated as 981085.XP_010108776.1. To evaluate the efficacy of this approach Figure 4B,C visualize the relative quantification of the 11-OH-THC molecule and the associated protein, respectively. The biosynthesis of cannabinoids both in vivo [34] and in vitro [35] systems is a focus of much research and we hope that the further development of this tool may enable researchers to more rapidly identify the proteins involved in these pathways.

## 3. Materials and Methods

### 3.1. Samples

A table of samples analyzed to date are described in Supplemental Table S1. All samples were obtained by Think20Labs under the guidelines of the Maryland Medical Cannabis Commission regulations in accordance with a temporary license granted under Code of Maryland Regulations 10.62.33 [36]. A recent study described the optimization of digestion conditions for the proteomic analysis of *Cannabis* flowers and performed similar experiments as the ones described here [31]. Vendor instrument files from that recent study are available at the MASSIVE data repository as MSV00083191. MGF files for all data described in this study may be downloaded from www.CannabisDraftMap.org.

### 3.2. Sample Preparation

Multiple variations on protein extraction and digestion were tested, based on the highest percent recovery of peptides per milligram of starting plant material by use of an absorbance (562 nm) assay for tryptic peptides (Pierce BCA Protein Assay Kit Cat# 23225) (data not shown). The final sample method was based on the filter-aided sample preparation method (FASP, Expedeon SKU:44250) [37]. Briefly, 1 mg of fresh plant flower was flash frozen at −80 °C for 20 min. The cell walls were disrupted by striking the flash frozen material once with a stainless-steel hammer. The material was then placed in a solution of 150 µL of 5% SDS and 0.2% DTT and heated at 95 °C for 10 min in a heating block to reduce and linearize the proteins. The temperature was then reduced to room temperature on ice. One hundred and fifty microliters of 8 M urea/50 mM TrisHCl was added to the mixture. Detergent removal, protein cysteine alkylation and sample cleanup for digestion was performed according to the FASP protocol. All reagents were obtained from Expedeon BioSciences. Proteins were digested with Trypsin (Promega) reconstituted in 25 mM Ammonium Bicarbonate in a 1:50 ratio (Trypsin: Protein) for 16 h at room temperature. Digested peptides were released by centrifuging the FASP chamber at 13,000× *g* for 10 min with peptides eluting into a new 1.5 mL centrifuge tube. An additional 75 µL of the 25 mM Ammonium bicarbonate was added and the elution was repeated. The peptides were completely dried by vacuum centrifugation (SpeedVac 3000× *g*, 3 hr.). Peptides were resuspended in 20 µL of 0.1% trifluoroacetic acid for either desalting or for high pH reversed phase fractionation. Peptides were quantified by absorbance using a peptide specific kit (Pierce, Cat # 23275).

### 3.3. Peptide Fractionation

Peptide fractionation followed two specific experimental designs. The first laying out the generation of highly fractionated peptides from 4 specific plant materials, which we will refer to as the green plant analysis experiment. The four samples were: commercial female flowers from an Indica dominant strain, the same from a Sativa dominant strain, followed by the leaves and stems from a hybrid male plant. Approximately 50 micrograms of peptides from each sample were fractionated with the Pierce high pH peptide fractionation kits (Cat # 84868) into 8 separate fractions by manufacturer protocol. A cartoon describing this process is shown in Supplemental Figure S1.

The second experiment focused on 8 commercially-available flower material and utilized an HPLC based fractionation strategy described in Supplemental Figure S2. Approximately 50 µg of peptide from each sample were combined and subjected to high resolution fractionation and followed a recent protocol [7], with the exception that an Accela 1250 pump (Thermofisher Scientific) was utilized for gradient delivery. Ninety-six fractions were collected using this method and every 8th sample was concatenated to produce 12 fractions as described previously [38].

### 3.4. LC-Mass Spectrometry Analysis

All fractionated and single shot samples were analyzed identically on a Thermo Scientific EasyNLC 1200-ESI-Q Exactive HF-X system. Briefly, 4 µg of peptides were loaded into a 4 cm trap column and eluted with an optimized gradient on a 100 cm monolithic 75 µm column. Eluting peptide masses were acquired at 120,000 resolution followed by the fragmentation of the most abundant eluting peptides with HCD fragmentation at 27 eV. Fragmented peptides were acquired at 15,000. Although this system is capable of higher scan speed, a higher resolution MS/MS was utilized in order to obtain more confident identification and localization of PTMs. The top 15 most abundant ions were selected for fragmentation with a 150 ms maximum ion injection time for each MS/MS scan. Dynamic exclusion was utilized allowing each ion to be fragmented once, any ion within 5 ppm of the matched ion was excluded from fragmentation for 60 s, or approximately 2.2× the peak width. 

### 3.5. Peptide and Protein Identification

**Figure 5:**
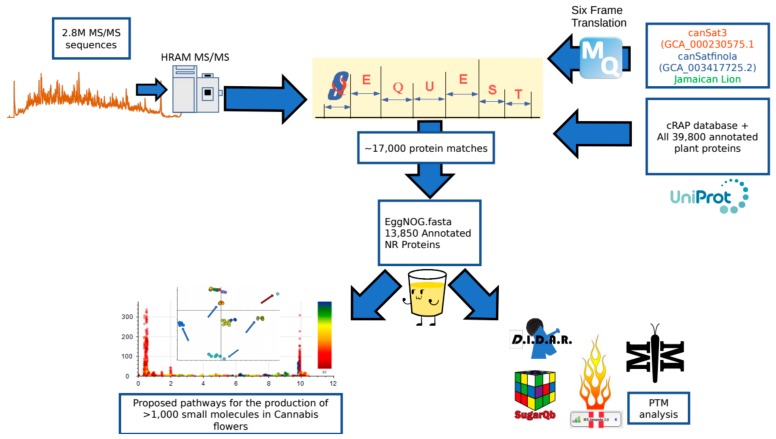
Peptide and protein identification pipeline. Two point eight million spectra obtained on a high resolution mass spectrometer were searched against a search space consisting of a six frame translation of three reference genomes as well as the cRAP FASTA and a complete collection of all green plant proteins hosted by UniProt. The 17,000 SEQUEST identifications were then processed with the eggNOG mapper program to annotate the identifications according to sequence orthology of the Viridiplantae database. This subsequent fasta could then be utilized to search the raw data for post-translational modifications using a variety of tools and identify pathways correlating to the small molecule profile of the plant.

An overview of the data processing pipeline and all input is demonstrated in Figure 5. At the beginning of this project, no fully annotated protein FASTA existed for any *Cannabis* species. Classical proteomics workflows require a reference theoretical protein database from which to construct matches from MS1 and MS/MS spectral data. In lieu of this we utilized two sources of information for identifying MS/MS spectra. As less than 600 annotated sequences for *Cannabis* exist in the UniProt library, a custom UniProt/SwissProt database consisting of every manually annotated sequence from green plants was used. In conjunction with this, the three highest quality genome sequences available in the literature [17,27] were subjected to 6-frame translation in house using the MaxQuant v1.6.3.3 [39] software suite to create theoretical protein sequences that accurately match the material being analyzed. This exercise resulted in a proteogenomic FASTA that contained theoretical sequences and arbitrarily assigned alphanumeric identifiers. This FASTA allows for initial analysis of genomic and post-translational modification data, but has limited value for downstream biological interpretation. For initial analysis, all data processing was performed in Proteome Discoverer 2.2 (PD) (Thermo Fisher) using the SEQUEST, Percolator and Minora algorithms. The proteogenomic FASTA was crudely reduced during database import in PD according to manufacturer default settings. SEQUEST and Percolator generate identity and confidence scored peptide spectral matches (PSMs). Multiple consensus workflows were used within PD to assemble the PSMs into peptide groups, protein database matches, and finally non-redundant proteins groups using the principle of strict parsimony as defined by the vendor software defaults. All settings utilized in the data processing to the generation of PSMs and the Consensus steps that reduce these matches to protein group identifications are described in Supplemental Table S2.

### 3.6. Generation of the EggNOG Annotated Protein FASTA

The 6-frame translated FASTA contained approximately 43.4 million potential protein sequences. Of these, 86,944 had at least one PSM uniquely matched to the proteogenomic FASTA or the UniProt Viridiplantae FASTA file and 58,309 were found to be non-redundant by sequence. All candidate protein sequences with at least one PSM were utilized to generate an annotated FASTA in the following manner. All proposed proteins sequences were exported from Proteome Discoverer 2.2 in FASTA format. To reduce and annotate this file, the eggNOG-mapper program [40] (http://eggnogdb.embl.de/#/app/emapper) was utilized with DIAMOND mapping mode with annotations utilizing any orthologs from the Viridiplantae database online as of the date of utilization (07/04/2019) using all orthologs and non-electronic terms. The returned files contained 30,988 putative sequence annotations. A total of 5735 entries had significant homology under the default server parameters for assignment to a specific gene. Using these settings, 796 sequences could not be assigned a significant match to the database for functional annotation. Further investigation will be necessary to determine if these proteins are artifacts of data processing or sequences uniquely present in these plants. The remaining 24,457 sequences were assigned a protein accession and functional annotation based on sequence homology. In order to preserve a unified format required for Proteome Discoverer, the gene name was replaced with the phrase “gene not found” when the best annotation was by protein function. The eggNOG annotation file and resulting FASTA were merged using an in-house generated script. The final annotated FASTA was compiled with the FASTA database utilities tool in Proteome Discoverer 2.2 and the compiled database was uploaded into the program, resulting in a final annotated database with 13,850 non-redundant annotated protein sequences. Throughout this manuscript we will refer to this as the eggnog FASTA.

### 3.7. Spectral Library Generation

The high-resolution MS/MS spectra were searched with PD 2.1 using the SEQUEST algorithm and eggNOG FASTA file using the same settings as described above and in Supplemental Table S2. The resulting .pdresult file was imported into the Skyline [41] 64-bit environment (version 4.1.0.1869) and converted to a spectral library according to the default parameters for Orbitrap high resolution MS/MS spectra [42]. The output spectral library can facilitate both targeted and Data Independent Analysis of plant proteins and is available for download at www.CannabisDraftMap.org.

### 3.8. Chromosome Alignment

A recent re-analysis of the CanSat3 genome [43] aligned the sequences into ten separate chromosome files [27]. The Protein Marker node in Proteome Discoverer was used in four rounds of reprocessing of the consensus workflow to develop a metric of the number of identified protein entries in this study that are products of each chromosome. Four rounds were necessary due to a limitation in the software that allows a maximum of 3 separate FASTA sequences to be used for output marking. Reiterations of this analysis were repeated to ensure that the chromosomes grouped in each re-analysis was an independent variable and did not affect localization output (data not shown). Protein information, representing both potential redundancies and unique protein groups were obtained. Using an exact match approach, 3421 proteins could be confidently mapped to one or more chromosomes. Cannabis has 10 pairs of chromosomes. Using this approach alignment is only possible to matching pair, not individual chromosome. The results are plotted in Supplemental Figure S5.

### 3.9. Identification and Validation of Potential Post-Translational Modifications (PTMs)

The MetaMorpheus open source software package v0.0.301 (MM, https://github.com/smith-chem-wisc/MetaMorpheus) was used for the indiscriminate identification of post translational modifications [23]. The unannotated FASTA file was used for MM analysis using the default workflows for Recalibration, GPTMD, Search and Post Processing [22,23] using default parameters. A resulting output file is Supplemental Table S4. To further confirm and visualize the presence of the most abundant PTMs, lysine acetylation and serine/threonine phosphorylation, the IMP-PD 2.1 (pd-nodes.org) was utilized within Proteome Discoverer. The workflow consisted of MSAmanda 2.0 operating with 5 ppm MS1 and 15 ppm MS/MS tolerance. The search was performed with eggNOG FASTA v.1.0, the Viridiplantae UniProt FASTA and the common lab contaminant database, cRAP. Static modification of carbamidomethylation of cysteine, with dynamic modifications of methionine oxidation, lysine acetylation and phosphorylation of serine and threonine were all enabled. The ptmRS algorithm [44] was used for confidence of site localization. All consensus workflow settings matched those described for the SEQUEST searches, with the exception that the localization of any PTM with 50% likelihood or greater was allowed for visualization. The .pdresult output file was visualized in MS2Go v1.4.7 (www.pd-nodes.org) according to default parameters. Files were filtered at the PSM level for lysine acetylation and phosphorylation of serine/threonine, respectively.

### 3.10. Gene Ontology Analysis for Green Plant Material

The fractionated files from the green plant material experiments were processed against the Viridiplantae UniProt FASTA in order to obtain gene identifiers compatible with downstream gene ontology (GO) analysis. The complete table of these results is provided as Supplemental Table S8 with GO assignments provided by the Protein Center Annotation node in Proteome Discoverer 2.2. Further visualizing was performed by selecting the proteins that differed by greater than 2.0 fold and by inserting these gene identifiers into the ShinyGO tool (V.0.61) [16]. ShinyGO networks were generated from the tool’s included network for *Arabidopsis thaliana* KEGG pathways. Figure 2 is a representative image of two differential networks.

### 3.11. Correlation Analysis of Small Molecules and Proteins

We have recently described the identification of approximately 1000 distinct small molecule features present in extractions from mature cannabis flowers [33]. Six samples used in this previous study were also used for the proteomic study described herein. These individual files were processed in PD 2.2 using SEQUEST searched against the eggNOG FASTA and utilizing the Minora algorithm for relative label free quantification according to manufacturer default settings. Quantification values were derived from pairwise analysis at the peptide group level. Abundance ratios were averaged when two or more peptide groups were observed for the protein. The resulting file contained 3661 protein groups and is provided as Supplemental Table S10.

A correlation analysis was then performed on the small molecule features and the label free quantification results utilizing Python (v3.7.3) along with the Pandas (v0.25.1) and Scipy (v.1.3.1) packages (https://github.com/jenkinsc11/probocor). For each small molecule that was identified, the changing areas of the features between samples were directly compared to the protein abundance variation. Pearson and Spearman correlation values were calculated along with their respective p-values. If either correlation analysis had the arbitrary p-value cut-off of less than or equal to 0.05, the small molecule–protein quantitation change between samples was flagged as having a possibly statistically significant correlation and compiled into a list for further investigation. Protein and small molecule abundances were manually extracted from highly correlating molecules using the Xcalibur 4.0 software. 

### 3.12. Scaffold Files for Relative Quantification

In order to generate relative quantification results across all files, the MSF result files from Proteome Discoverer were imported into Scaffold 4.0 (Proteome Sciences) using the default input for quantification with spectral counts. For the green plant experiments, all fractions for each plant matter starting material were combined into one. MSF file by Proteome Discoverer and each .MSF was imported into Scaffold as a separate “BioSample.” For the flower specific experiments, each individual file was processed as a separate.MSF file and imported as its own BioSample. The Scaffold .sf3 files were exported as Excel files and are presented as Supplemental Tables S6 and S7, respectively.

### 3.13. Graph Generation

The UpSetR package was used for the comparison of proteome to genome sequencing files using both the webhosted ShinyApp (https://gehlenborglab.shinyapps.io/upsetr/) as well as the full package within RStudio 1.0.143. Supplemental figures were generated in the Pacific Northwest National Laboratory Venn Diagram 1.5.5 tool (https://omics.pnl.gov/software/venn-diagram-plotter) as well as with GGPlot2 [45] within RStudio.

## 4. Conclusions

We have performed the first comprehensive proteomic analysis of *Cannabis* plants, the first step towards our goal of developing a multi-omics biochemical map of these plants. From the samples analyzed to date and described herein, we have peptides that correspond to 17,269 open reading frames from the genomic data present in the literature. Traditional proteomics workflows rely on the existence of annotated theoretical protein FASTA files derived from annotated genomes. At the beginning of this project, no fully annotated protein FASTA file existed for any *Cannabis* plant.

We have developed a pipeline by which any material with both “next generation” sequencing and shotgun proteomics data may be used to generate theoretical protein FASTA files directly, thus circumventing the need for annotated genomes entirely. The output of this pipeline is the most comprehensive protein FASTA for *Cannabis* constructed to date, consisting of 13,850 nonredundant sequences with putative annotations. With the creation of this large species-specific database we can now utilize traditional proteomics tools for the identification and quantification of proteins from these plants. Furthermore, we have identified diverse chemical modifications on proteins central to metabolism that appear linked to terpene and cannabinoid production in the plant. We have found that *Cannabis* plants possess numerous post-translationally modified proteins, namely lysine acetylation sites and phosphorylation of threonine and serine, as well as evidence of extensive protein glycosylation of currently unknown site localization and glycan chain structure. The analysis and role of these PTMs may be of interest to future research as lysine acetylation appears to be involved in the production of Cannabis molecules of commercial and medical interest. In addition, correlating proteomics measurements with phenotypic data such as chemical profiles will provide a valuable resource for producers and concerned consumers. This is a critical next step in the advancement of the medical applications of *Cannabis*. An overall summarization of the results from this study are found in Table 1 below.

To facilitate further study of these plants, we have made our FASTA database, annotated spectra and spectral libraries publicly available with the release of this manuscript, along with other resources at www.CannabisDraftMap.org.

## 5. Future Goals

We aim to identify the function of PTMs in these plants, specifically how these modifications correlate to the production of secondary metabolites. Multiple alternative algorithms and approaches may be used to further refine, improve and annotate all resources described in this study and investigation into these approaches are currently underway. Furthermore, it is our belief that big data is only useful if it is made available to the largest possible audiences. We will continue to work on clarifying our results and making these available to the wider community through improved software and interfaces, with a specific focus on improving and expanding on the spectral libraries available here to enable robust targeted and data independent acquisition analysis of these plants. 

All results described in this study and updated results will be made freely available at www.CannabisDraftMap.org.

## 6. Significance Statement

Until recently laws in North America have restricted nearly all research on Cannabis plants. Currently only a few hundred proteins from the plant have been sequenced. We have performed the first in depth proteogenomic study of cannabis plant materials resulting in the annotation of over 13,800 proteins as well as collected chemical information on over 1000 small molecules produced by medicinal plants. We demonstrate for the first time that protein acetylation and glycosylation are abundant PTMs in cannabis plants and may be involved in the regulation and production of small molecules of commercial interest. All results and resources enabling further analysis into these plants are available at www.CannabisDraftMap.org.

## Figures and Tables

**Table 1:** An overview of the progress to date.

Category of Data	Number in 2019 Upload
Protein Sequenced	17,269
Protein Annotated	13,929
Proteins with homologous 3D structures	964
Acetylation sites Mapped	584
MS/MS Spectra Acquired	1.40 × 10^7^
MS/MS Spectra Searched	2.40 × 10^6^
MS/MS Spectra with Evidence of Glycosylation	3.50 × 10^5^
Skyline Spectral Library	43,612 annotated spectra
Gene Coding Regions Annotated	13,850
Small Molecule Features Isolated	1050
Small Molecules Identified	535

## Supplementary Materials

Supplementary materials can be found at https://www.mdpi.com/1422-0067/21/3/965/s1.

## References

[1] Zhang Y., Fonslow B.R., Shan B., Baek M.C., Yates J.R. (2013). Protein analysis by shotgun/bottom-up proteomics. Chem. Rev..

[2] Yates J.R., Eng J.K., McCormack A.L., Schieltz D. (1995). Method to Correlate Tandem Mass Spectra of Modified Peptides to Amino Acid Sequences in the Protein Database. Anal. Chem..

[3] Kim M.S., Pinto S.M., Getnet D., Nirujogi R.S., Manda S.S., Chaerkady R., Madugundu A.K., Kelkar D.S., Isserlin R., Jain S. (2014). A draft map of the human proteome. Nature.

[4] Wilhelm M., Schlegl J., Hahne H., Gholami A.M., Lieberenz M., Savitski M.M., Ziegler E., Butzmann L., Gessulat S., Marx H. (2014). Mass-spectrometry-based draft of the human proteome. Nature.

[5] Hebert A.S., Richards A.L., Bailey D.J., Ulbrich A., Coughlin E.E., Westphall M.S., Coon J.J. (2014). The one hour yeast proteome. Mol. Cell. Proteomics.

[6] Kelstrup C.D., Jersie-Christensen R.R., Batth T.S., Arrey T.N., Kuehn A., Kellmann M., Olsen J.V. (2014). Rapid and deep proteomes by faster sequencing on a benchtop quadrupole ultra-high-field orbitrap mass spectrometer. J. Proteome Res..

[7] Bekker-Jensen D.B., Kelstrup C.D., Batth T.S., Larsen S.C., Haldrup C., Bramsen J.B., Sorensen K.D., Hoyer S., Orntoft T.F., Andersen C.L. (2017). An Optimized Shotgun Strategy for the Rapid Generation of Comprehensive Human Proteomes. Cell Syst..

[8] Lössl P., Waterbeemd M., Heck A.J. (2016). The diverse and expanding role of mass spectrometry in structural and molecular biology. EMBO J..

[9] Witze E.S., Old W.M., Resing K.A., Ahn N.G. (2007). Mapping protein post-translational modifications with mass spectrometry. Nat. Methods.

[10] Silva A.M.N., Vitorino R., Domingues M.R.M., Spickett C.M., Domingues P. (2013). Post-translational modifications and mass spectrometry detection. Free Radic. Biol. Med..

[11] Ghazalpour A., Bennett B., Petyuk V.A., Orozco L., Hagopian R., Mungrue I.N., Farber C.R., Sinsheimer J., Kang H.M., Furlotte N. (2011). Comparative analysis of proteome and transcriptome variation in mouse. PLoS Genet..

[12] Raharjo T.J., Widjaja I., Roytrakul S., Verpoorte R. (2004). Comparative proteomics of cannabis sativa plant tissues. J. Biomol. Tech..

[13] Sun L., Xu Y., Bai S., Bai X., Zhu H., Dong H., Wang W., Zhu X., Hao F., Song C.P. (2019). Transcriptome-wide analysis of pseudouridylation of mRNA and non-coding RNAs in Arabidopsis. J. Exp. Bot..

[14] Prasad T.S.K., Mohanty A.K., Kumar M., Sreenivasamurthy S.K., Dey G., Nirujogi R.S., Pinto S.M., Madugundu A.K., Patil A.H., Advani J. (2017). Integrating transcriptomic and proteomic data for accurate assembly and annotation of genomes. Genome Res..

[15] Conway J.R., Lex A., Gehlenborg N. (2017). UpSetR: An R package for the visualization of intersecting sets and their properties. Bioinformatics.

[16] Ge S.X., Jung D. (2018). ShinyGO: a graphical enrichment tool for animals and plants. bioRxiv.

[17] McKernan K.J., Helbert Y., Kane L.T., Ebling H., Zhang L., Liu B., Eaton Z., McLaughlin S., Kingan S., Baybayan P. (2020). Sequence and annotation of 42 cannabis genomes reveals extensive copy number variation in cannabinoid synthesis and pathogen resistance genes. bioRxiv.

[18] Jenkins C., Orsburn B. (2019). The First Publicly Available Annotated Genome for Cannabis plants. bioRxiv.

[19] Zirpel B., Kayser O., Stehle F. (2018). Elucidation of structure-function relationship of THCA and CBDA synthase from Cannabis sativa L.. J. Biotechnol..

[20] Jenkins C., Norris A., O’Neill M., Das S., Andresson T., Orsburn B. (2018). Reporter Ion Data Analysis Reduction (R.I.D.A.R) for isobaric proteomics quantification studies. bioRxiv.

[21] Li Q., Shortreed M.R., Wenger C.D., Frey B.L., Schaffer L.V., Scalf M., Smith L.M. (2017). Global Post-Translational Modification Discovery. J. Proteome Res..

[22] Millikin R.J., Solntsev S.K., Shortreed M.R., Smith L.M. (2018). Ultrafast Peptide Label-Free Quantification with FlashLFQ. J. Proteome Res..

[23] Solntsev S.K., Shortreed M.R., Frey B.L., Smith L.M. (2018). Enhanced Global Post-translational Modification Discovery with MetaMorpheus. J. Proteome Res..

[24] Finkemeier I., Laxa M., Miguet L., Howden A.J.M., Sweetlove L.J. (2011). Proteins of diverse function and subcellular location are lysine acetylated in Arabidopsis. Plant Physiol..

[25] Booth J.K., Bohlmann J. (2019). Terpenes in Cannabis sativa – From plant genome to humans. Plant Sci..

[26] Paul Zolg D., Wilhelm M., Schmidt T., Médard G., Zerweck J., Knaute T., Wenschuh H., Reimer U., Schnatbaum K., Kuster B. (2018). Proteometools: Systematic characterization of 21 post-translational protein modifications by liquid chromatography tandem mass spectrometry (lc-ms/ms) using synthetic peptides. Mol. Cell. Proteomics.

[27] Laverty K.U., Stout J.M., Sullivan M.J., Shah H., Gill N., Holbrook L., Deikus G., Sebra R., Hughes T.R., Page J.E. (2019). A physical and genetic map of Cannabis sativa identifies extensive rearrangements at the THC/CBD acid synthase loci. Genome Res..

[28] Singh C., Zampronio C.G., Creese A.J., Cooper H.J. (2012). Higher energy collision dissociation (HCD) product ion-triggered electron transfer dissociation (ETD) mass spectrometry for the analysis of N-linked glycoproteins. J. Proteome Res..

[29] Hoffmann M., Pioch M., Pralow A., Hennig R., Kottler R., Reichl U., Rapp E. (2018). The Fine Art of Destruction: A Guide to In-Depth Glycoproteomic Analyses—Exploiting the Diagnostic Potential of Fragment Ions. Proteomics.

[30] Toghi Eshghi S., Yang W., Hu Y., Shah P., Sun S., Li X., Zhang H. (2016). Classification of Tandem Mass Spectra for Identification of N- and O-linked Glycopeptides. Sci. Rep..

[31] Vincent D., Rochfort S., Spangenberg G. (2019). Optimisation of protein extraction from medicinal cannabis mature buds for bottom-up proteomics. Molecules.

[32] Singh S.A., Winter D., Kirchner M., Chauhan R., Ahmed S., Ozlu N., Tzur A., Steen J.A., Steen H. (2014). Co-regulation proteomics reveals substrates and mechanisms of APC/C-dependent degradation. EMBO J..

[33] Jenkins C., Orsburn B. (2019). Application of Global Metabolomics to the Identification of Complex Counterfeit Medicinal Products. bioRxiv.

[34] Luo X., Reiter M.A., D’Espaux L., Wong J., Denby C.M., Lechner A., Zhang Y., Grzybowski A.T., Harth S., Lin W. (2019). Complete biosynthesis of cannabinoids and their unnatural analogues in yeast. Nature.

[35] Wróbel T., Dreger M., Wielgus K., Słomski R. (2018). The application of plant in vitro cultures in cannabinoid production. Biotechnol. Lett..

[36] Mead A. (2017). The legal status of cannabis (marijuana) and cannabidiol (CBD) under U.S. law. Epilepsy Behav..

[37] Shen S., An B., Wang X., Hilchey S.P., Li J., Cao J., Tian Y., Hu C., Jin L., Ng A. (2018). Surfactant Cocktail-Aided Extraction/Precipitation/On-Pellet Digestion Strategy Enables Efficient and Reproducible Sample Preparation for Large-Scale Quantitative Proteomics. Anal. Chem..

[38] Navarrete-Perea J., Yu Q., Gygi S.P., Paulo J.A. (2018). Streamlined Tandem Mass Tag (SL-TMT) Protocol: An Efficient Strategy for Quantitative (Phospho)proteome Profiling Using Tandem Mass Tag-Synchronous Precursor Selection-MS3. J. Proteome Res..

[39] Weisser H., Nahnsen S., Grossmann J., Nilse L., Quandt A., Brauer H., Sturm M., Kenar E., Kohlbacher O., Aebersold R. (2013). An automated pipeline for high-throughput label-free quantitative proteomics. J. Proteome Res..

[40] Huerta-Cepas J., Forslund K., Coelho L.P., Szklarczyk D., Jensen L.J., Von Mering C., Bork P. (2017). Fast genome-wide functional annotation through orthology assignment by eggNOG-mapper. Mol. Biol. Evol..

[41] MacLean B., Tomazela D.M., Shulman N., Chambers M., Finney G.L., Frewen B., Kern R., Tabb D.L., Liebler D.C., MacCoss M.J. (2010). Skyline: An open source document editor for creating and analyzing targeted proteomics experiments. Bioinformatics.

[42] Egertson J.D., MacLean B., Johnson R., Xuan Y., MacCoss M.J. (2015). Multiplexed peptide analysis using data-independent acquisition and Skyline. Nat. Protoc..

[43] van Bakel H., Stout J.M., Cote A.G., Tallon C.M., Sharpe A.G., Hughes T.R., Page J.E. (2011). The draft genome and transcriptome of Cannabis sativa. Genome Biol..

[44] Taus T., Köcher T., Pichler P., Paschke C., Schmidt A., Henrich C., Mechtler K. (2011). Universal and confident phosphorylation site localization using phosphoRS. J. Proteome Res..

[45] Wickham H. (2009). ggplot2.

