# Supplementary figures and images for: The Cannabis Proteome Draft Map Project
Source: Int J Mol Sci. 2020 Jan 31;21(3):965. doi: 10.3390/ijms21030965 (PMC7037972; doi:10.3390/ijms21030965)

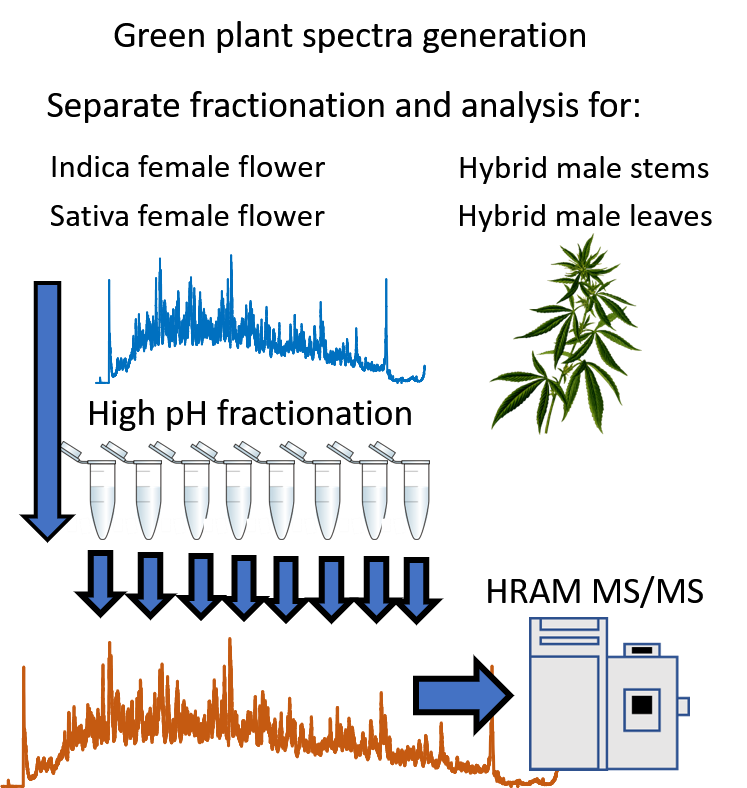

Supplement: Supplementary file 1 [file ijms-21-00965-s001.zip › ijms-654092-SI-to conversion/ijms-654092-Supplemetary Figures/FigureS1.tif]

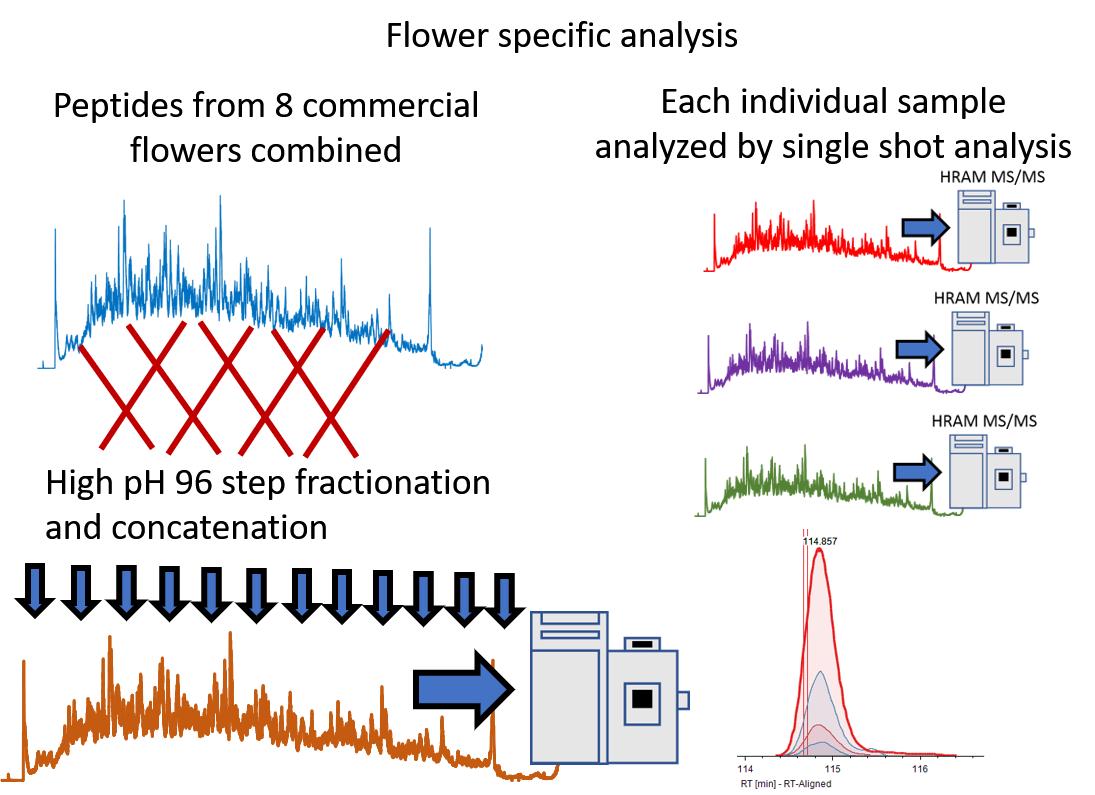

Supplement: Supplementary file 1 [file ijms-21-00965-s001.zip › ijms-654092-SI-to conversion/ijms-654092-Supplemetary Figures/FigureS2.tif]

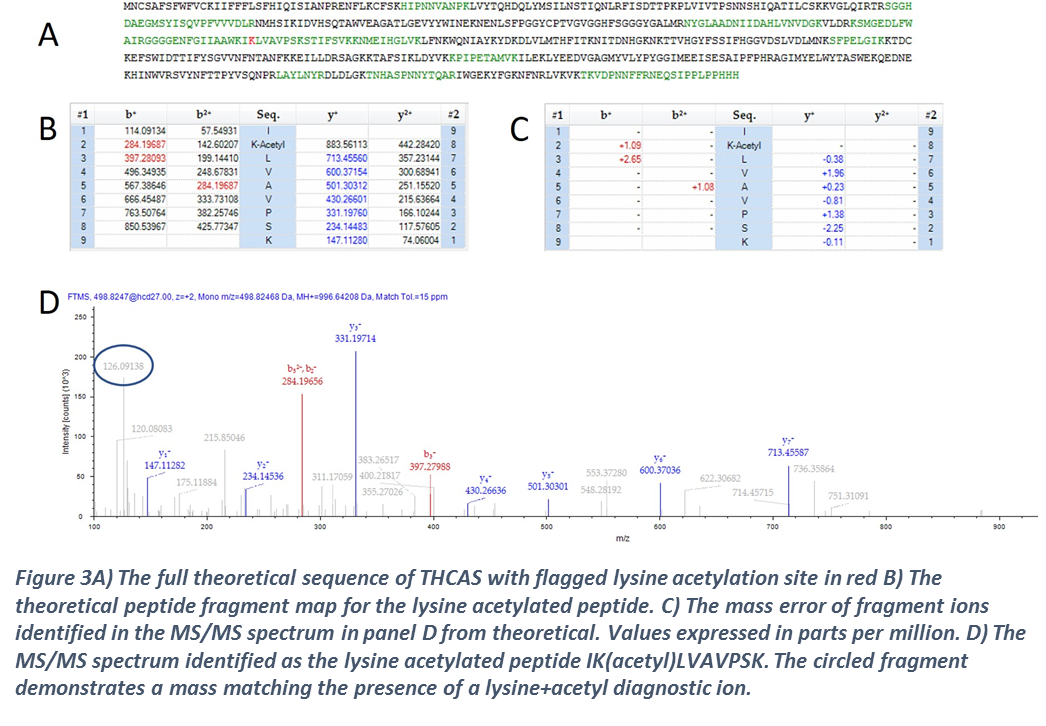

Supplement: Supplementary file 1 [file ijms-21-00965-s001.zip › ijms-654092-SI-to conversion/ijms-654092-Supplemetary Figures/FigureS3.tif]

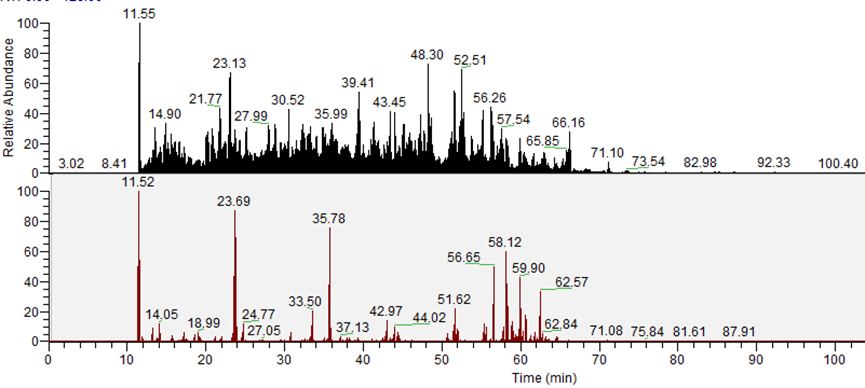

Supplement: Supplementary file 1 [file ijms-21-00965-s001.zip › ijms-654092-SI-to conversion/ijms-654092-Supplemetary Figures/FigureS4.tif]

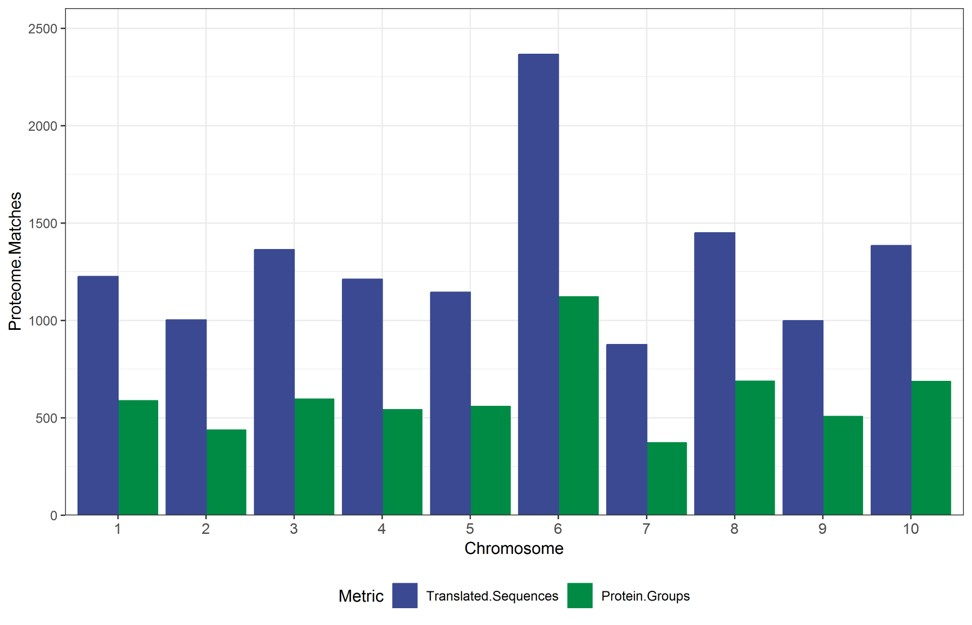

Supplement: Supplementary file 1 [file ijms-21-00965-s001.zip › ijms-654092-SI-to conversion/ijms-654092-Supplemetary Figures/FigureS5.tif]
